# Cognition and inflammation

**DOI:** 10.1192/j.eurpsy.2026.10345

**Published:** 2026-07-31

**Authors:** G. Sachs, A. Erfurth

**Affiliations:** 1 Medical University of Vienna; 2 1st Department of Psychiatry and Psychotherapeutic Medicine, Klinik Hietzing, Vienna, Austria; 3 University of Muenster, Muenster, Germany

## Abstract

**Abstract:**

The association between elevated inflammation levels and cognitive alterations in inflammation-associated diseases supports the notion that inflammation plays a role in modulating cognition under physiological circumstances. Dysregulation of the immune system could therefore contribute to the cognitive changes observed in severe psychiatric disorders such as psychotic, unipolar and bipolar affective disorders (1).

A meta-analysis of inflammation and cognition in schizophrenia (2) revealed very modest associations between peripheral inflammatory biomarkers, such as C-reactive protein (CRP), and cognition. Elevated CRP levels were associated with impaired performance in all cognitive domains except verbal fluency. Similarly, a meta-analysis of inflammation and cognition in individuals with affective and psychotic disorders (3) revealed a weak association between global cognitive ability and pro-inflammatory indicators. Peripheral markers such as CRP have been shown to be highly state-dependent in mood and psychotic illnesses, suggesting that they are more relevant as biomarkers for episodic symptoms. A study of patients presenting with their first episode of schizophrenia found that an increased neutrophil-to-lymphocyte ratio was negatively associated with cognitive function (4).

In our ongoing study (5), we assess cognitive impairments in acute psychiatric inpatients diagnosed with psychotic, bipolar or depressive disorders, using the German version of the SCIP (6). Our sample comprises routinely collected clinical data from adult psychiatric patients living in a specific catchment area who were previously indicated for acute, inevitable admission following an emergency assessment. Data examining the effect of CRP values and the neutrophil-to-lymphocyte ratio on cognitive function in our sample of acute patients is shown.

Fourrier C, Singhal G, Baune BT. Neuroinflammation and cognition across psychiatric conditions. CNS Spectr. 2019;24(1):4-15.

Bora E. Peripheral inflammatory and neurotrophic biomarkers of cognitive impairment in schizophrenia: a meta-analysis. Psychological Medicine. 2019;49(12):1971-1979.

Morrens M, et al. The relationship between immune and cognitive dysfunction in mood and psychotic disorder: a systematic review and a meta-analysis. Mol Psychiatry. 2022;27(8):3237-3246.

Liang J, et al. Neutrophil/lymphocyte ratio and cognitive performances in first-episode patients with schizophrenia and healthy controls. Prog Neuropsychopharmacol Biol Psychiatry. 2024;135:111092.

Maihofer EIJ, Sachs G, Erfurth A. Cognitive Function in Patients with Psychotic and Affective Disorders: Effects of Combining Pharmacotherapy with Cognitive Remediation. J Clin Med. 2024;13(16):4843.

Sachs G, Lasser I, Purdon SE, Erfurth A. Screening for cognitive impairment in schizophrenia: Psychometric properties of the German version of the Screen for Cognitive Impairment in Psychiatry (SCIP-G). Schizophr Res Cogn. 2021;25:100197.

**Disclosure of Interest:**

None Declared

